# n and p type character of single molecule diodes

**DOI:** 10.1038/srep08350

**Published:** 2015-02-10

**Authors:** Vinícius Claudio Zoldan, Ricardo Faccio, André Avelino Pasa

**Affiliations:** 1 Max Planck Institute of Microstructure Physics, Halle, Germany; 2 Laboratório de Filmes Finos e Superfícies, Universidade Federal de Santa Catarina, Florianópolis, Brazil; 3 Cryssmat-Lab and Centro NanoMat, Facultad de Química, Universidad de la República, Montevideo, Uruguay

## Abstract

Looking for single molecule electronic devices, we have investigated the charge transport properties of individual tetra-phenylporphyrin molecules on different substrates by ultrahigh-vacuum scanning tunneling microscopy and spectroscopy and by first-principles calculations. The tetra-phenylporphyrins with a Co atom (Co-TPP) or 2 hydrogens (H_2_-TPP) in the central macrocycle when deposited on Cu_3_Au(100) substrates showed a diode-like behavior with p and n type character, respectively. After removing the central hydrogens of H_2_-TPP molecule with the STM tip an ohmic behavior was measured. The rectifying effect was understood from the theoretical point of view by assuming for Co-TPP HOMO conduction and for H_2_-TPP LUMO conduction, both selectively elected by the hybridization of states between molecule and substrate surface.

The control and understanding of transport properties of single molecules is the ultimate aim of molecular electronics. An envisaged component nowadays is a molecular diode, where a single molecule acts as a rectifier blocking the charge transport in one direction and allowing the flow in the opposite one. Molecular diodes were predicted by Aviram and Ratner in 1974^1^ and achieved experimentally in 2005 as single-molecule diode by Elbing and co-authors^2^, when an asymmetric designed molecular rod, with two weakly couple conjugate units and Au-S covalent bonds at the terminals, presented diode-like curves, and by Zhao et. Al.^3^ where a C_59_N molecule in a double-barrier tunnel showed a rectifying effect. Later on, Tao and co-workers^4^ have reported on rectifying and stability of an asymmetrical molecule with controlled orientation covalently bound to two gold electrodes.

However, despite the advances of the recent years, it is still a challenge to reach stable systems and reproductive for single molecule measurements. For example, molecular wiring for transport measurements, using the STM-break junction setup, have been intensively used to characterized a large number of molecules^5^,^6^. It is one way to study and understand its properties, but at the same time, requires a very high repetition of the experiments to get reliable results.

Recently, Lei and co-authors^7^ demonstrate by Low Temperature Scanning Tunneling Microscopy (LT-STM) measurements and Density Functional Theory (DFT) simulations a orbital-selective rectifier effect for single cobalt phthalocyanine (CoPc) molecules adsorbed on graphene-covered Ru(0001) surface. More recently, Batra and coauthors^8^ showed tunable rectification in single molecules devices using a symmetric, conjugated molecular backbone with a gold-carbon covalent bonds at one end and sulfide linkers at the other end.

In this work, the charge transport properties of individual tetra-phenylporphyrin molecules on different substrates were studied by Ultrahigh-Vacuum Scanning Tunneling Microscopy and Scanning Tunneling Spectroscopy (UHV-STM/STS) measurements. The molecules used were tetra-phenylporphyrins (TPP) with a Co atom (Co-TPP) or 2 hydrogens (H_2_-TPP) in the central macrocycle. Porphyrins are particularly interesting molecules due to their variety of functional properties, which are relevant for biological and artificial systems^9^. This versatile molecule can be synthesized with a wide diversity of meso-substituents and can also host different metals in the core macrocycle^10^,^11^,^12^. These facts make the porphyrins great candidates for single molecule devices, in addition to the property that the electron transport is determined by the local electronic structure of the analyzed nanoscale region (molecule and contacts)^13^. What also makes the electrical responses very sensitive to atomic manipulations of the molecules^14^.

Many of the STM studies of Porphyrin molecules have used substrates with (111) orientation, which allows room temperature deposition of molecular monolayers^11^,^15^. In the present work, three different substrates are used to test different interaction between molecule and substrate, Ag(111) where monolayer of molecules on the surface are formed^11^, Cu_3_Au(100) where deposition of single molecules is obtained at room temperature, and Cu_3_N-Cu(110) where the deposition of single molecules occurs even at room temperature and the partial decoupling between molecule and substrate is observed^16^. As a main result, in this combined experimental and theoretical study, we observed that the tetra-phenylporphyrins with a Co atom (Co-TPP) or 2 hydrogens (H_2_-TPP) in the central macrocycle, when deposited on Cu_3_Au(100) substrates, show diode-like behavior with p and n type character,respectively. We were also able to manipulate the H_2_-TPP electrical response by removing the central hydrogens.

## Results and Discussion

**Figure 1:**
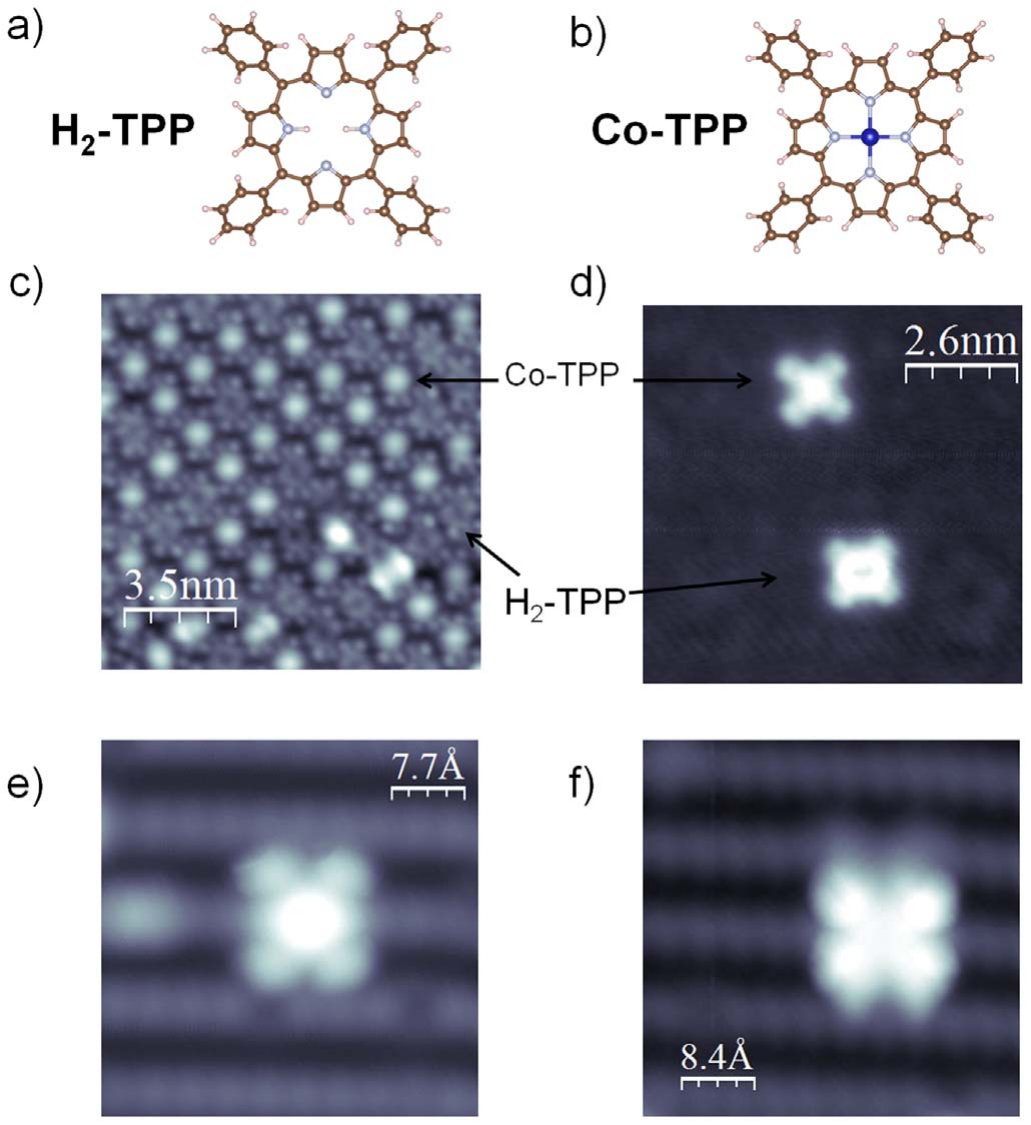
(a) and (b) molecular structure of the H_2_TPP and Co-TPP molecules, respectively, (c) monolayer of Co-TPP and H_2_-TPP molecules on Ag(111), (d) single molecules of Co-TPP and H_2_-TPP on Cu_3_Au(100), and (e) Co-TPP and (f) H_2_-TPP on Cu_3_N.

Figures 1a and 1b display the molecular structure of the H_2_TPP and Co-TPP molecules, respectively. Our studies start with the deposition of a mixture of these molecules on Ag(111), which is a well known substrate for the deposition of porphyrins^11^. On this substrate, at room temperature even for low coverage agglomerates with monolayer thickness are formed. Due to the singular contribution of the occupied and unoccupied electronic states obtained at negative and positive bias voltages, respectively, a strong bias dependence on the apparent size and height of the molecule is observed in constant current STM images^11^. Figure 1c is a high resolution STM image of a layer comprising both molecules, Co-TPP and H_2_-TPP, on Ag(111) obtained for a tunneling current of 2.0 nA and bias voltage of −0.20 V. For this energy (voltage) we can easily see the difference between the molecules, where for Co-TPP what is visible is the Co atom in the center of the porphyrin macrocycle and the four phenyl rings symmetrically distributed around it, while for the H_2_-TPP the center of the macrocycle is empty.

We have done also deposition on Cu_3_Au (100) substrates, where the strong interaction between molecule and substrate allows the deposition of single molecules at room temperature. Figure 1d shows the constant-current STM topography obtained at 0.30 V and 2.0 nA for individual molecules of Co-TPP (top) and H_2_-TPP (bottom) on the Cu_3_Au(100) surface. For these STM parameters, similar to the Ag(111) case, the molecules present analogous cross shape with nearly the same size and apparent height, with the exception that for Co-TPP molecule the Co atom is at higher apparent position while for the H_2_-TTP the highest apparent places are evidenced for the porphyrin macrocycle. However, it should be noticed here that the relative apparent heights and shapes of H_2_TPP and Co-TPP porphyrins are strongly bias dependent on this substrate, bias dependence that is evidenced in the IxV curves discussed below.

Figure 1 shows also experiments conducted on a cupper nitrate monolayer on Cu(110), aiming an electronic decoupling between the molecule and the substrate^16^. Individual molecules of Co-TPP (0.5 nA and −1.0 V) and H_2_-TPP (1.0 nA and −0.2 V) can be undoubtedly differentiated on Cu_3_N-Cu(110) surface as can be seen in Figure 1e and 1f, respectively.

**Figure 2:**
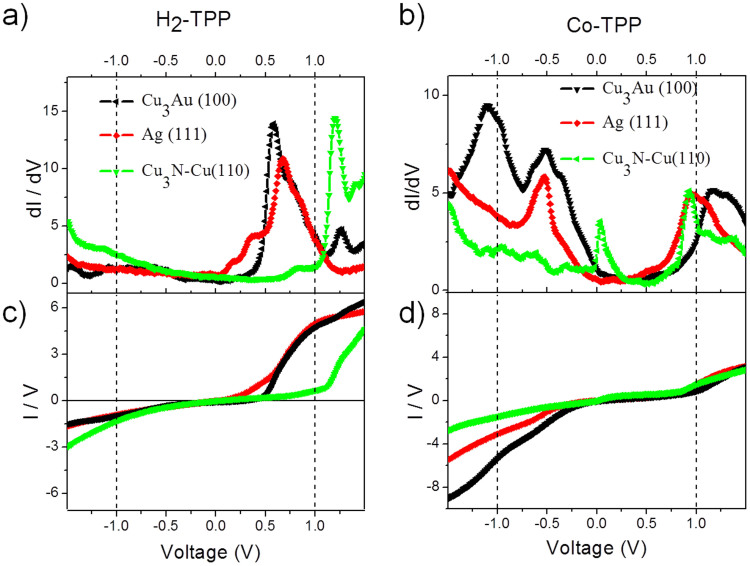
(a) and (b) dI/dV spectra taken at the center of the single H_2_-TPP and Co-TPP molecules on different substrates. (c) and (d) I–V curves corresponding to the dI/dV measurements.

Figures 2a and 2b show the dIxdV curves taken with the STM tip over the center of the molecules deposited on the three different substrates, Cu_3_Au(100), Ag (111), and Cu_3_N-Cu(110). For H_2_-TPP molecule, Figure 2a, for all substrates, only the LUMO (lowest unoccupied molecular orbital) is observed in the range of voltage between −1.5 and 1.5 V. For Ag(111) and Cu_3_Au(100) substrates, the LUMO peak is located at energies lower that 1.0 V, whereas the peak for the Cu_3_N(100) surface is above 1.0 V and much better defined. For the Co-TPP molecule, Figure 2b, both highest occupied molecular orbital (HOMO) and LUMO are observed for all substrates. However, for the Cu_3_N substrate the HOMO peak is not defined and for Cu_3_Au a strong intensity of the occupied molecular orbital when compared with the other substrates is observed together with a shift of the LUMO to energies above 1.0 V. The peak at the Fermi level for the Cu_3_N surface could be attributed to Kondo effect, as mentioned in a previous work^16^.

In Figures 2c and 2d are displayed the IxV curves obtained for the H_2_-TPP and Co-TPP molecules. In Figure 2c, a molecular rectifying behavior is observed, since a non-linear dependence of the current versus voltage is observed. The electrical characteristic is typical of a pn junction or a metal/n-type semiconductor Schottky junction. For Co-TPP molecule, Figure 2d, the non-linear characteristic is typical of np or metal/p-type junctions. At a first glance, the n and p type features are attributed to LUMO peaks for H_2_-TPP molecules and HOMO peaks for Co-TPP molecules observed in Figure 2a and 2b, respectively. In case of H_2_-TPP the onset voltage is dependent on the substrate, where a value above 1.0 V is observed for the Cu_3_N-Cu(110) surface, in agreement with the shift in LUMO position. For the Co-TPP molecules, the rectification ratio is higher for molecules on Cu_3_Au(100), in accordance with the higher intensity observed for the occupied molecular orbitals for this substrate.

Thus, for a better understanding of the above results, we performed a careful theoretical and experimental study of the absorption and electron transport processes for both molecules on Cu_3_Au(100) substrate. The diode like response was quantified, as usually^26^, by calculating the rectification ratio (*RR*(*V*) = |−*I*(*V*)/*I*(−*V*)|) at a specific value of applied potential. We have also checked the dependence of this ratio for different tip-molecule distances. From I–V curves obtained with tunneling current of 2 nA (tunnel gap set for a sample bias voltage of 0.65 V), rectification ratios of 8.3 and 4.7 for H_2_-TPP and Co-TPP were obtained at |*V*| = 0.75 V, respectively. By increasing the distance of the tip to the surface (0.2 nA and 0.65 V of sample bias voltage), rectification ratios of 3.6 and 6.1 were obtained for Co-TPP and H_2_-TPP molecules, respectively. In this case, the magnitude of the ratio showed an additional distinction between the molecules, i. e., by increasing the tip distance the rectification action decreased for Co-TPP and increased for H_2_-TPP.

**Figure 3:**
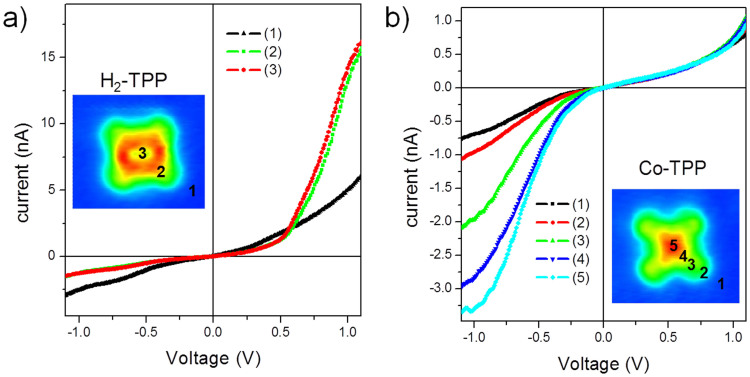
Current-voltage characteristic curves measured at different positions over the (a) H_2_-TPP and (b) Co-TPP molecules absorbed on Cu_3_Au (100) substrate. The insets are STM images with the indication of the position were the curves were acquired. The STM topographic image were obtained at 0.30 V and 2.0 nA for both molecules. The I–V curves were obtained at a −0.55 V sample bias voltage and with a 2.0 nA set current for the H_2_-TPP, while for Co-TPP the parameters are −0.60 V and 0.3 nA.

The diode-like effect was also observed by moving laterally the tip over the molecule on the Cu_3_Au (100) substrate. As shown in Figure 3a, H_2_-TPP single molecules presented no significant dependence of the IxV curves on the positioning of tip on top of the center or on top of the porphyrin macrocycle, positions 3 and 2, respectively. This suggests that H_2_-TPP when deposited on Cu_3_Au has the LUMO orbital uniformly distributed throughout the porphyrin macrocycle as extended states. On the other hand, for Co-TPP, Figure 3b, a strong dependence of the IxV curves was observed on the position of the tip above the molecule, from position 2 to 5, indicating that this effect comes from the Co atom, similarly to what was observed by Lei et. al.^7^ for a cobalt phthalocyanine molecule on top of a graphene monolayer on Ru substrate. They also observed a np character in their samples. As a reference, an I–V curve of the metallic substrate is also added in Figure 3a and 3b, with the expected linear dependence as a function of voltage (ohmic behavior), measured in position 1 in both cases.

**Figure 4:**
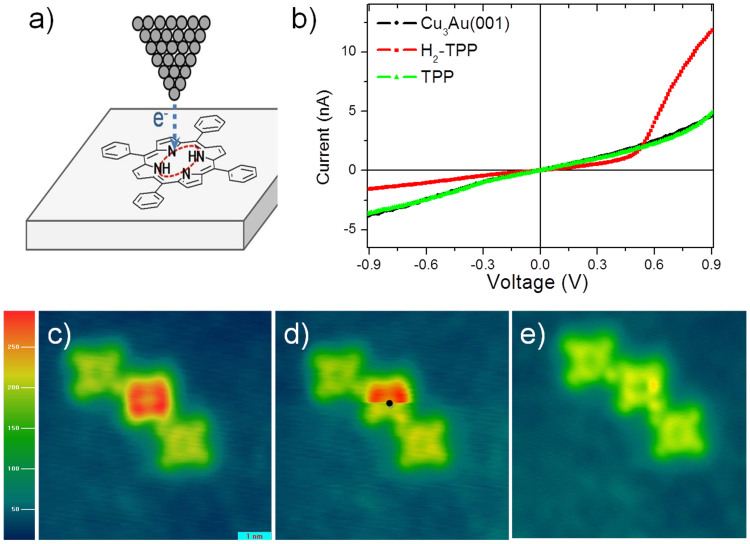
STM tip-induced dehydrogenation of a single H_2_-TPP molecule. (a) Schematic illustration showing the dehydrogenation induced by the STM current. In this case, when applying 2.1 V with the STM tip on the center of the H_2_-TPP molecule the two hydrogen atoms are dissociated. (b) Typical I–V curves measured at the center of the H_2_-TPP molecule before and after the dehydrogenation. The I–V curve of the Cu_3_Au substrate is shown for comparison. (c–e) Sequence of STM images of the dehydrogenation process of the H_2_-TPP molecules on Cu_3_Au. (c) Three molecules, 2 TPP and 1 H_2_-TPP in the center. (d) The black dot illustrates the position when voltage is applied and the Hs were dissociated. (e) STM image after the dehydrogenation. STM topographic image were obtained at 0.54 V and 2.0 nA.

We have performed also atomic manipulation of the molecule H_2_-TPP on Cu_3_Au, as shown in Figure 4, aiming for tuning the rectifying action of the single molecules. Figure 4a shows a schematic illustration of the dehydrogenation process induced by the STM current. The hydrogen atoms were removed by applying 2.1 eV, following the procedure presented in reference 14, with the STM tip on the center of the H_2_-TPP molecule. Figure 4b displays typical I–V curves measured at the center of the H_2_-TPP molecule before and after the dehydrogenation. The I–V curve of the Cu_3_Au substrate is also shown for comparison. The TPP molecule, that is, the H_2_-TPP molecule without the 2 hydrogen atoms presents a linear response similar to the Cu_3_Au substrate. Sequences of theoretical dIxdV curves (not shown) taken with the TPP molecule with 2, 1 and 0 hydrogen atoms showed as a general trend that by removing the hydrogen, the LUMO peaks increase in intensity and shift to higher energies and the HOMO peaks only increase significantly in intensity.

Figures 4c to 4e display the sequence of STM images of the dehydrogenation process of the H_2_-TPP molecules. In 4c, three molecules are observed but only the one in the middle is still with the H atoms. In 4d the black dot added to the image illustrates the exactly position of the tip when the voltage was applied for dissociation of the hydrogen atoms. Figure 4e is just the STM image after the dehydrogenation. STM topographic images were obtained at 0.54 V and 2.0 nA.

When molecules are absorbed on conductive surfaces, the interaction with the surface electron density allows the contribution of different molecular orbitals to the tunneling current through the molecule. The differences on the molecular orbitals and consequently on the apparent height of the molecules on the surface by STM can be observed in the Figure 4c to 4e. In this figure, after the process of dehydrogenation, the H_2_-TPP presents a variation of about 36% on its apparent height when the constant-current STM topography image is obtained at 0.54 V and 2.0 nA. After the dehydrogenation, the electrical curve of molecule followed the curve of the ohmic substrate, a result that could be explained by assuming a different molecule-substrate interaction promoted by the removal of the hydrogen atoms on the center of the porphyrin, as discussed below.

**Figure 5:**
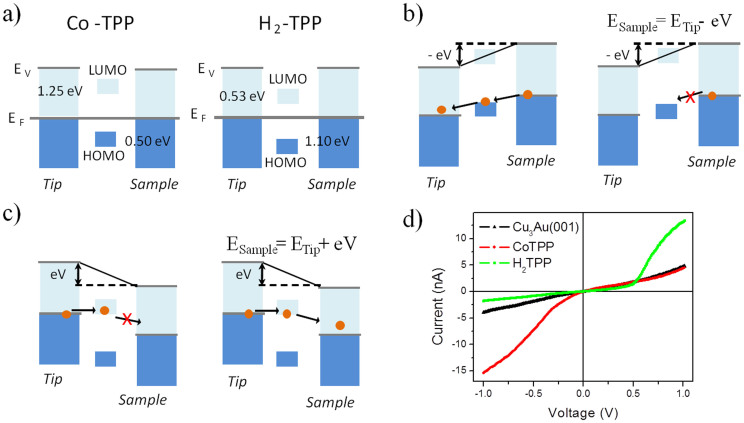
Representation of the conduction mechanism, through the molecule, for the sample–molecule–tip system on Cu_3_Au surfaces. (a) Energy diagram without applied bias voltage for the Co-TPP and H_2_-TPP molecules. (b–c) Complementary rectification effect due to HOMO conduction for Co-TPP and LUMO conduction for H_2_-TPP. (d) The experimental I × V curve obtained for the molecules on Cu_3_Au(100).

Figure 5a is an illustration of the band structure of the system formed by sample (substrate), molecule and tip at equilibrium. At left is shown the band structure for Co-TPP and at right for H_2_-TPP molecules both on Cu_3_Au surfaces. The relative positions of the HOMO and LUMO to the Fermi level were obtained from the calculation, which are in very good agreement with the experimental ones. Figure 5b and 5c are illustrations of the rectification mechanism of the molecules when the tip is positively or negatively biased relatively to the sample, respectively. For positive bias, electrons from the sample are transported to the tip through HOMO states of the Co-TPP molecule. For the H_2_-TPP molecule, besides the existence of HOMO states, no electron is injected as illustrated. For negative bias, electrons from the tip are transported to the H_2_-TPP molecule through LUMO states. However, no electrons from LUMO states are injected to states in the Cu_3_Au surface. The existence of HOMO and LUMO states from the molecules and states from the substrate surface are not a warranty of charge transport between tip and sample. The rectification mechanism presented in Figure 5b and 5c describes the electrical conduction observed experimentally in Figure 5d. The explanation for HOMO conduction (injection of electrons to HOMO states from the surface) for Co-TPP and LUMO (injection of electrons from the LUMO states to the surface) for H_2_-TPP molecules is related to the hybridization of molecule and surface states as will be described below from the theoretical calculations.

**Figure 6:**
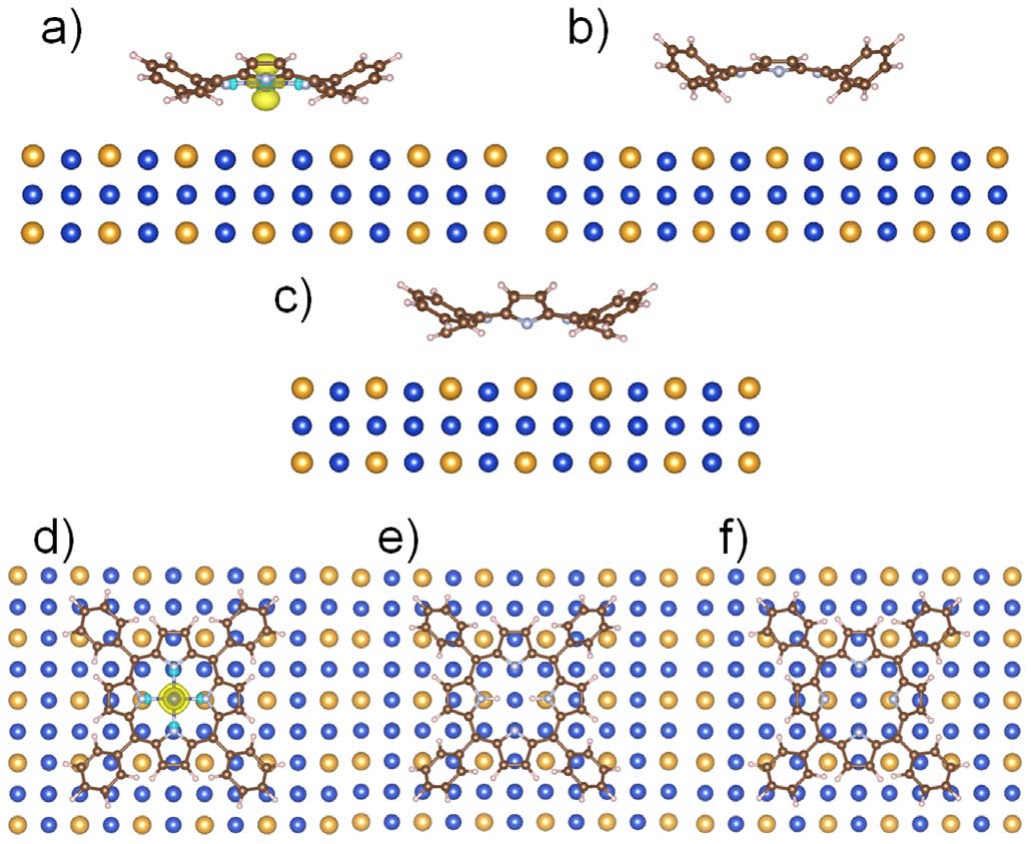
Final geometries projected along [100] direction for (a) Co-TPP, (b) H_2_-TPP and (c) TPP; and projected along [001] direction for (d) Co-TPP, (e) H_2_-TPP and (f) TPP. The molecules are adsorbed on the Cu_3_Au(100) surface. Note: For Co-TPP, (a) and (d) images include the iso-surfaces for spin density, showing the S = 1/2 electronic configuration for Co(II) located at the Co-dz^2^ orbitals. The light blue and yellow colors represent the up and down spins, respectively.

The side and top views for Co-TPP, H_2_-TPP and TPP molecules adsorbed on the Cu_3_Au(100) are represented on Figure 6. For the Co-TPP molecule adsorbed on the Cu_3_Au(100) surface, see top view structural model in Figure 6d, we observe that after the geometrical optimization the four nitrogen atoms in the porphyrin macrocycle are located above two Cu and two Au atoms in a symmetric way. Consequently, the Co atom in the center of the porphyrin is sitting on the top of a Cu atom. Similar absorption has been obtained for the H_2_-TPP and TPP where the empty center of the molecule is located exactly above a Cu atom, as displayed in Figure 6e and 6f.

Regarding the electronic structure of Co-TPP, we obtained the spin S = 1/2 solution for Co(II) ions, as expected for this molecule^16^. This is graphically presented in Figure 6a and 6d, where the S = 1/2 is located at the Co-dz^2^ atomic orbital. The molecule to surface distance corresponds to d ~ 3.8 Å, obtained as an average distance between N atoms and the proximate Cu atoms from the Cu_3_Au surface. The molecule to surface distance for H_2_-TPP and TPP, correspond to d ~ 4.6 Å and 3.8 Å, respectively.

The calculated distances are representative of the interaction of the molecules with the substrate. The presence of cobalt atom in the Co-TPP is relevant, since it interacts strongly with the surface. In case of TPP, two hydrogen atoms are missing enhancing the reactivity of the under-coordinated N atoms, pointing directly to the metallic atoms of the surface and inducing important changes in the core of the porphyrin. For H_2_-TPP molecule, the interaction is weaker when compared to the other two cases. This behavior is expected, since there is no metallic atom at the center of the porphyrin and the N atoms are saturated by the two hydrogen atoms.

**Figure 7:**
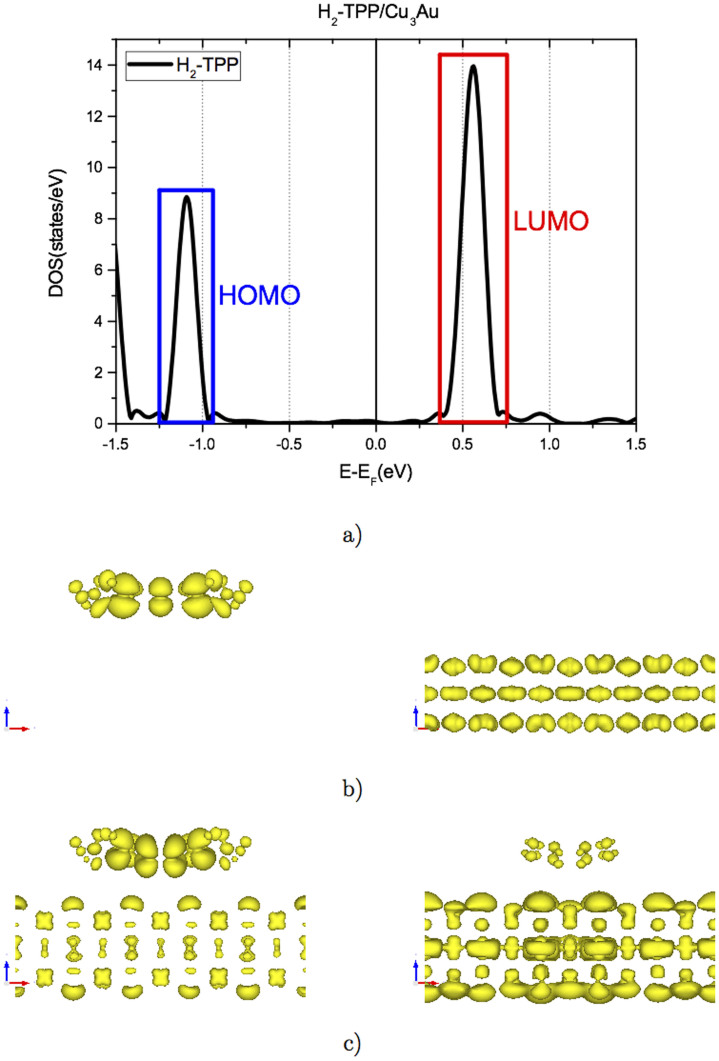
(a) Projected Density of States (PDOS) for H_2_-TPP, Cu_3_Au surface is not included. Orbital decomposed charge density for HOMO (b) and LUMO (c) from H_2_-TPP, at Γ-point, showing the hybridization between π-states from TPP and d-sates from Cu_3_Au just in the case of LUMO. Note that in the case of HOMO, there is no relevant hybridization between surface and molecule. Only two charge densities are shown for HOMO and LUMO from a total of 11 bands in each case, for monoelectronic wavefunction calculated considering the molecule/substrate atomic model of Figure 6b.

In reference to the electronic structure, the H_2_-TPP molecule promoted an enhancement of the current at positive bias in the IxV experiment (see Figure 2c). This experimental result can be well understood in terms of the DFT calculations. First of all, we have obtained the theoretical atomic projected density of states (PDOS), presented in Figure 7a, that confirms the presence of molecular states in the range of −1.5 a 1.5 eV. The HOMO and LUMO for H_2_-TPP are located at −1.1 eV and 0.55 eV, respectively. In particular, the LUMO's position is in very good agreement with the experimental dI/dV curves. Nevertheless, the HOMO orbitals were not observed experimentally, but this fact can be explained on the basis of orbital hybridization between surface and molecule as we will describe below.

In the case of HOMO, we selected an energy region between −1.25 and −0.90 eV shown by the blue rectangle in Figure 7a. This region is composed by a total of 11 bands and to understand the interaction between the molecule and the surface, we have obtained the charge density associated to each monoelectronic wavefunction from calculations considering the molecule/substrate atomic model of Figure 6b. For all HOMO 11 bands the charge density results can be summarized in two cases, i. e. one that contains only states from the molecules and the other that contains orbitals only from the substrate, as represented in Figure 7b. This calculation allows us to conclude that in the HOMO region of the H_2_-TPP molecules there is no relevant hybridization between molecule and surface, thus reducing the STM tunneling current in that energy region in agreement with the results displayed in Figure 5d.

The LUMO region, red rectangle in Figure 7a, is also composed of 11 bands. The orbital density charge calculations showed three bands showing important hybridization between molecule and surface, involving π-states and d-sates, respectively. Two of the three bands are showed in Figure 7c. This hybridization is extremely relevant when considering the results of STM experiments in Figure 5d, since a considerable increase in the tunneling current is observed for the LUMO position of H_2_-TPP molecule on Cu_3_Au.

For the TPP molecule, the removal of the two hydrogen atoms resulted in significant changes in the plane of the porphyrin core modifying the nature of the chemical bonding and the corresponding molecular orbitals. The projected density of states (PDOS), result not shown, displays states located in the vicinity of the Fermi level as non-bonding molecular orbitals for TPP. In the energy range of −1.0 to 1.0 eV three peaks are present and centered at 0.10, −0.55 and −0.95 eV. The first two peaks when decomposed in their corresponding bands and the obtained monoelectronic wavefunctions show contributions from TPP or Cu_3_Au surface, without any relevant hybridization between molecule and surface. In the same way, the PDOS peak locate at −0.95 eV, composed by eleven bands, shows only one band that has an incipient hybridization between TPP and Cu_3_Au. The molecular orbitals with a major contribution of π-states from the under coordinated nitrogen atoms of the dehydrogenated H_2_-TPP are located down to −1.0 eV. This result is expected, since the geometrical distortions indicated that the interaction of these N atoms with surface is stronger when compared to H_2_-TPP. These states are involved in a more intense interaction producing a shift to more negative energies. The changes in electronic structure of the TPP molecule when compared to H_2_-TPP, explains the ohmic behavior for voltages between −1.0 and 1.0 V, as observed in Figure 4b.

**Figure 8:**
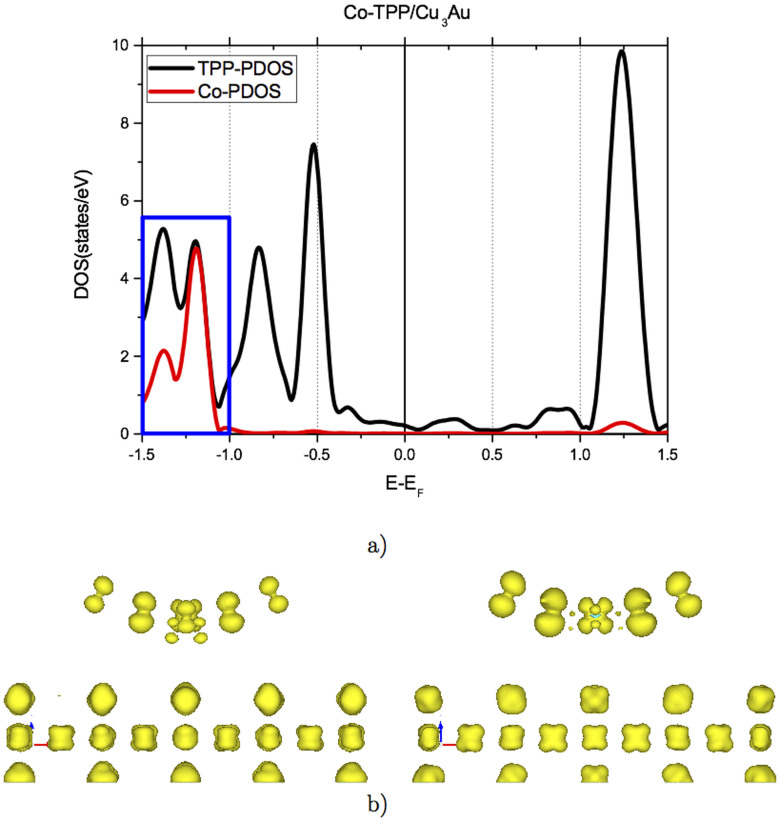
(a) Projected Density of States (PDOS) for Co-TPP, separated in Co (red) and TPP (black) contributions, Cu_3_Au is not included for clarity. (b) Charge density associated to 2 representative monoelectronic wavefunctions, calculated using the structural model of Figure 6a, showing hybridization between π-states from TPP and d-sates from Co with d-states from Cu_3_Au for energies bertween −1.5 and −1.0 eV.

In the case of Co-TPP, the molecule promotes an enhancement in the current for negative bias in the IxV experiments (see Figure 5d). The projected density of states (PDOS) displayed in Figure 8a confirms the presence of molecular states in the range of −1.5 to 1.5 eV. The HOMO is located at −0.53 eV and corresponds to orbitals with strong contribution from π-states from the TPP. In the region between −1.5 and −1.0 eV, blue rectangle in Figure 8a, there are 8 bands with contributions from mixed π-TPP and d-Co states, in particular from Co-d_xz_ and Co-d_yz_ orbitals. The LUMO is located at 1.25 eV comprising mainly π-states from TPP and very low contribution from d-states of cobalt. Once again, there is a very good correspondence between theory and experimental dI/dV curve of Figure 2b. In reference to the enhancement of the current in IxV curves in Figure 2d, the major differences arise close to −1.0 eV. At this energy there is a relevant hybridization between surface and molecule with d-states from Co-d and Cu_3_Au, as graphically represented in Figure 8b. In this figure we plotted the charge density associated to 2 representative monoelectronic wavefunctions, calculated using the structural model of Figure 6a. Important contributions from Co-dxz and Co-dyz orbitals in addition to π-states and d-states from the CoTPP and Cu_3_Au are observed. While the presence of molecular states at energies between −0.25 and −1.0 eV could explain the STM current at low negative applied voltages, Co-d states improves the hybridization between the TPP molecule and Cu_3_Au surface. This fact is very relevant for the STM current at bias voltages more negative than −1.0 V. For positive voltages no STM current is observed, since the projected density of states is very low, as shown in Figure 8a for energies in the range of 0 to 1.0 eV.

In conclusion, the p and n diode-like character was seen in H_2_-TPP and Co-TPP molecules on Cu_3_Au surfaces, respectively. By removing the 2 hydrogen atoms of H_2_-TPP the ohmic behavior was observed. The charge transport through the system tip-molecule-surface could be explained for the H_2_-TPP molecule by the hybridization between π-states from TPP and d-sates from Cu_3_Au, and for the molecule Co-TPP the hybridization between π-states from TPP and d-sates from Co with d-states from Cu_3_Au.

## Methods

### Experimental Section

The experiments were carried out in a low-temperature STM system operated at 4.6 K, consisting of three separated ultrahigh vacuum (UHV) chambers used for substrate preparation, molecules deposition and STM analysis. The single crystalline samples were cleaned by repeated cycles of Ar^+^ ion sputtering and annealing steps and the Cu_3_N-Cu(110) monolayer was growth follow the previous procedure^16^.

The tetra-phenylporphyrin molecules were purified by vacuum sublimation and deposited on all surfaces with the substrates kept at room temperature. IxV and Spectroscopic measurements were performed using the lock-in technique with the modulation of the bias voltage at 5 mV, where positive sample bias voltage corresponds to unoccupied states, negative sample bias voltage to occupied states and zero Volts represents the position of the Fermi level. All spectra taken from the molecules were checked by measuring the dI/dV spectrum of the substrate before and after measuring the molecule to avoid peaks due to tip effects.

### Computational section

The computational methodology is analogous to our previous and related work for CoTPP interacting with Cu_3_N-Cu(110) surfaces^16^. The theoretical method is based on First Principles – Density Functional Theory^17^,^18^. The simulations were performed using the ab-initio program VASP (Vienna ab-initio simulation program)^19^,^20^,^21^,^22^ developed in the Institut für Material Physik of the Universität Wien. The PBE^23^ generalized gradient approximation (GGA) functional has been used, and the projector-augmented wave method (PAW)^24^,^25^ has been employed to treat the atomic cores. The precision setting for the VASP calculations, which sets to the one that corresponds to a global plane-wave, was an energy cutoff of 400 eV. The k-point sampling corresponds to a 2 × 2 × 1 grid.

The calculations were performed for CoTPP/Cu_3_Au, H_2_TPP/Cu_3_Au, TPP/Cu_3_Au in a periodic structure, were the unit cell parameters correspond to a = b = 19.3 Å and c = 23.1 Å. The Cu_3_Au surface was modeled using a three-layer slab, with the bottom layer fixed in their calculated optimized bulk positions, and the top two layers were allowed to fully relax. The vacuum separation between the Cu_3_Au slabs was 19.2 Å, leaving about 14.8 Å between molecule and the back of the next periodic image of the slab. Dipole corrections were included in the [001] directions, and Van der Waals interactions were neglected in all the cases. The adsorption geometries were obtained by a full optimization, placing the CoTPP molecule above the surface and allowing all atoms and the top two layers of the slab to fully relax until all the forces on the atoms were less than 0.01 eV/Å.

## Author Contributions

V.C.Z. carried out the experiments and analyzed the data, R.F. performed all the theoretical calculations, A.A.P. conceived and supervised the project, and V.C.Z., R.F. and A.A.P. wrote the paper.
